# Sociodemographic and Regional Inequalities in Antenatal Care (ANC) in Brazil

**DOI:** 10.7759/cureus.90775

**Published:** 2025-08-22

**Authors:** Narayani M Rocha, Wanessa da S Almeida, Maria do C Leal, Celia L Szwarcwald

**Affiliations:** 1 Institute of Scientific and Technological Communication and Information in Health, Oswaldo Cruz Foundation, Rio de Janeiro, BRA; 2 National School of Public Health Sergio Arouca, Oswaldo Cruz Foundation, Rio de Janeiro, BRA

**Keywords:** access to healthcare, antenatal care, barriers, brazil, quality of care, social inequalities

## Abstract

Background: Despite improvements in antenatal care (ANC) coverage in Brazil over the years, social inequalities persist, compromising the quality and effectiveness of the care provided to pregnant women. This study aimed to estimate the prevalence of pregnant women by adequacy of prenatal care according to sociodemographic characteristics and regions of residence.

Methods: Using data from the 2019 Brazilian National Health Survey, we analyzed 2,875 women aged 15 years or older who had given birth in the two years preceding the survey. Based on the Ministry of Health guidelines, ANC was categorized as inadequate (less than six consultations or not having started in the first trimester of pregnancy) and in three different levels of adequacy, from least to most adequate. To identify the main determinants of high-quality ANC, a multivariate logistic regression model was applied, using sociodemographic and geographic variables as predictors.

Results: Prevalence of women with inadequate ANC was 22.8%. Adequacy proportions of levels 1, 2, and 3 were 77.2, 51.8, and 42.4%, respectively. According to the results of the multivariate logistic regression model, women living in the North and Northeast regions were less likely to receive adequate ANC compared with those in the South (OR = 0.60 and OR = 0.65, 95% CI 0.39-0.93 and 0.44-0.95, respectively; p < 5%). Women living in urban areas compared with rural areas (OR = 1.64; 95% CI 1.20-2.26, p < 1%), as well as those with a partner or spouse (OR = 1.41; 95% CI 1.04-1.91, p < 5%), were significantly more likely to receive adequate ANC. Women with primary education showed less adequate ANC (OR = 0.56; 95% CI 0.35-0.88, p < 5%), as did younger women. Having been assisted by a doctor during pregnancy was significantly associated with better adequacy (OR = 1.72; 95%CI 1.29-2.29, p < 1%). The other variables (residence in the capital, race/skin color, and location of consultations) were not statistically significant.

Conclusion:  The findings emphasize the importance of monitoring the quality of ANC, especially to identify and meet the needs of the most vulnerable women during pregnancy.

## Introduction

Since the late 1990s, Brazil has undergone significant changes in terms of socioeconomic development and urbanization. In addition to economic growth, advances in healthcare and progress in primary care, particularly with the consolidation of the Family Health Strategy, have contributed to improved outcomes in child health [1]. Between 1990 and 2015, there was a 40% decrease in the infant mortality rate [2], with sharp declines in diarrheal diseases and lower respiratory infections [3]. High vaccination coverage achieved by the national immunization program led to a significant reduction in the incidence of vaccine-preventable diseases [4]. Furthermore, the country reached near-universal coverage of births attended by qualified professionals and achieved over 87% coverage of antenatal care (ANC), as measured by the percentage of women having six or more visits [5]. Although the progress in public policies aimed at promoting child health and development is undeniable, unresolved issues remain. Since 2015, a severe economic crisis in Brazil has led to increased poverty, threatening the achievement of the Sustainable Development Goals for child health and the reduction of inequality [6]. 

Brazil is a middle-income country with major socioeconomic inequalities, mainly due to difficulties in accessing and utilizing health services [7]. Thus, the main challenges facing the country are ensuring equal opportunities for access to health services at all levels and providing comprehensive and quality health care to all Brazilians, in accordance with the principles of the Unified Health System (SUS) [8].

During the pregnancy-puerperal cycle, ANC is an essential component of maternal and child health, playing a crucial role in preventing complications and achieving positive outcomes for both mothers and newborns [9]. In Brazil, ANC is part of primary healthcare and is offered free of charge through the SUS.  

According to the guidelines of the Ministry of Health (MoH), several recommendations are made to pregnant women to ensure quality care: ANC should begin in the first trimester; women should attend six or more appointments; undergo blood tests, HIV and syphilis testing; the baby's heartbeat should be checked, blood pressure measured, and uterine height monitored at each visit. All procedures carried out must be properly recorded in the Pregnant Woman’s Handbook, a fundamental tool to ensure continuity of care and facilitate referral and counter-referral processes among health services. Additionally, prior linkage of the pregnant woman to the healthcare facility where the delivery will occur is an essential strategy to prevent the need to seek care during labor, promoting greater safety and quality in obstetric care [10-13].  

Despite improvements in ANC coverage in Brazil over the years [5], inequalities still persist, compromising the quality and effectiveness of the care provided to pregnant women. In addition to issues related to the lack of quality in ANC, such as failures to comply with MoH recommendations, factors such as poverty, low educational attainment, inadequate housing, poor sanitation, and social exclusion significantly impact access to healthcare during pregnancy [2,14]. Given this scenario, it is essential to assess the performance of ANC in terms of quality of care. 

International studies have developed indices to measure the adequacy of ANC in different countries, based not only on coverage but also on the quality of services provided [15]. Among the proposals are the availability of health services and trained health personnel to provide appropriate care [16]. Strategies based on geospatial analysis to improve access to health services have also been used [17]. Other interventions have focused on health education and the empowerment of pregnant women to improve adherence to prenatal care and overcome potential pregnancy complications [18]. 

Some international guidelines on the quality of prenatal care differ from those followed in Brazil. Since 2016, the World Health Organization (WHO) has recommended at least eight prenatal consultations, while the Brazilian Ministry of Health has recommended six consultations as the minimum standard since 2011. However, similarities can also be identified, such as HIV and syphilis testing, considered by both the WHO and Brazil to be a central strategy for eliminating vertical transmission of HIV and syphilis [19]. 

Information on live births in Brazil has been publicly available since 1994 in the Live Birth Information System (SINASC). The data are based on the live birth certificate (DNV), a mandatory document at the health facility where the birth occurred. The system represents an important source of data for guiding policies for pregnant women and newborns at the municipal, state, and national levels. Regarding ANC, the system has only two indicators: number of consultations and trimester of initiation. For six or more consultations and initiation by the first trimester, ANC is considered adequate. In 2019, 67.4% of women received adequate prenatal care according to SINASC criteria. This percentage increased to 72.2% in 2023 [20]. 

Additional information comes from national surveys, such as the "Birth in Brazil Study", conducted initially in 2011-12 and subsequently in the period 2022-24, but the ANC results are still not available [12]. The survey includes data on prenatal care and clinical and obstetric examinations performed during consultations; clinical and obstetric history; current obstetric history; laboratory test results; prescription of nutritional supplements and medications; diagnoses and prescription of treatments for diseases or complications during pregnancy [21]. 

Another source of information is the National Program for Improving Access and Quality of Primary Care (PMAQ), which describes the temporal evolution of prenatal quality indicators in the primary health care from 2012 to 2018 [22]. 

Considering the Ministry of Health guidelines [10-13] and based on data from the 2019 National Health Survey, this study established criteria to assess the quality of prenatal care, classified into four levels (Inadequate, Level 1, Level 2, and Level 3). In view of the persistent inequalities in ANC quality care found in previous studies [2,14], the objectives of this study were to estimate the prevalence of pregnant women at each level of adequacy and investigate the associations of sociodemographic and geographic factors with the levels of ANC adequacy.

## Materials and methods

Information source  

The Brazilian National Health Survey (Pesquisa Nacional de Saúde in Portuguese - PNS) is a cross-sectional, nationwide, household-based study conducted by the MoH in partnership with the Brazilian Institute of Geography and Statistics (IBGE). It was carried out in the years 2013 and 2019, collecting a wide range of data to understand the health profile of the Brazilian population [23].  

The PNS sample is a subsample of the Master Sample from the Integrated Household Survey System of the IBGE and was selected through three-stage cluster sampling. In the first stage, the primary sampling units (PSUs), consisting of one or more census tracts, were randomly selected in each stratum of the IBGE Master Sample. In the second stage, a fixed number of households was randomly selected in each PSU. In the third stage, one resident aged 15 years or older was randomly selected to answer the individual questionnaire. Residents of special census tracts were excluded from the survey. The survey response rate was 93.6% [24].  

The 2019 PNS was approved by the National Research Ethics Committee (CONEP) in August 2019, under protocol number 3.529.376.  

In this study, data from the 2019 PNS were used, specifically from the ANC Module of the individual questionnaire, referring to ANC for the most recent childbirth. Women 15 years or older who had given birth in the two years prior to the survey, i.e., between July 28, 2017, and July 27, 2019, were included in the sample. 

 All analyses were conducted using IBM SPSS Statistics for Windows, Version 21 (Released 2012; IBM Corp., Armonk, New York, United States), accounting for the complex sampling design of the 2019 PNS.  

Variables  

To analyze sociodemographic factors, the following variables were selected: 14 levels of education (elementary, up to junior high school, up to senior high school, and complete college education); maternal age (15-19 years, 20-24 years, 25-29 years, 30-34 years, 35 years or older); race/skin color (white, black, mixed); living with a partner (yes or no); and per capita household income (up to one minimum wage or more than one minimum wage).  

The variables related to ANC considered in this study were: type of service where ANC was provided (public or private) and health professional responsible for most ANC visits (doctor or non-doctor).  

As for geographic factors, the following variables were considered: area of residence (urban or rural); residing in a state capital (yes or no); and macro-region of residence (North, Northeast, Southeast, South, Center-West). 

ANC adequacy indicators  

Considering the Ministry of Health guidelines [10-13] and on available data in the 2019 PNS, the following indicators were used to assess the quality of ANC: trimester of ANC initiation; number of ANC visits; HIV and syphilis testing; number of visits in which fetal heartbeat was monitored, blood pressure was measured, and uterine height was recorded; and whether the woman received guidance on the place of delivery [10]. Based on these criteria and using data from the 2019 PNS, ANC adequacy was classified into quality levels, as shown in Table 1. 

**Table 1:** Classification of the ANC adequacy, PNS, 2017-2019 Information Source: National Health Survey 2019 [23] ANC: Antenatal care

Levels of Quality	Variables
Level 1	ANC initiated by the first trimester and six or more visits.
Level 2	ANC initiated by the first trimester and six or more visits. Guidance on the place of delivery. Completion of HIV and syphilis testing.
Level 3	ANC initiated by the first trimester and six or more visits. Guidance on the place of delivery. Completion of HIV and syphilis testing. Fetal heartbeat monitoring, uterine height, and blood pressure checks at every visit.
Inadequate	Less than six consultations or did not start ANC in the first trimester of pregnancy.

Data analysis

To describe the sample, proportions of pregnant women were estimated for each category of the sociodemographic and geographic variables. The proportions were calculated considering the total number of women who answered the question in each category of the study variables, without adjusting for missing values. 

To analyze the contribution of each study variable to ANC adequacy, we presented the proportional distribution (%) of pregnant women by sociodemographic, geographic, and ANC-related characteristics according to the levels of ANC adequacy (Inadequate, Level 1, Level 2, and Level 3). To investigate the association between those factors with adequacy levels, Pearson's chi-square test was applied. Associations were considered statistically significant at a p-value < 0.05.  

To identify the main determinants of ANC adequacy (Level 3), a multivariate logistic regression model was used, with the independent variables being all categories of the study variables, except for per capita household income, excluded to avoid potential collinearity with education, as the two variables were significantly correlated (r = 0.59, 95% CI 0.56 - 0.62, p < 1%, where r is the correlation coefficient). Odds ratios (ORs) were estimated to test associations, with significance set at 5%.  

## Results

A total of 2,875 women aged 15 years or older who had given birth in the two years preceding the survey were analyzed. 

Regarding sociodemographic indicators, 41.7% of women had incomplete college education; 52.5% identified as mixed race; 77.8% lived with a partner; 74.4% had a per capita household income of up to one minimum wage; 70.3% received ANC in public services; and 28.8% were attended by physicians in most of their consultations. As for geographic characteristics, 84.3% lived in urban areas, and 79.1% did not reside in state capitals. According to Brazil’s macro-regions, 11.8% lived in the North, 29.6% in the Northeast, 36.9% in the Southeast, 12.5% in the South, and 9.2% in the Center-West (Table 2). 

**Table 2:** Proportional distribution (%) of pregnant women according to sociodemographic, antenatal care, and geographic characteristics (Brazil, PNS-2019) MW**: Minimum Wage in Brazil

Variable	n	%
Level of Education		
Up to Elementary School	558	19.4
Up to Junior High School	589	20.5
Up to Senior High School	1199	41.7
Complete College Education	529	18.4
Maternal Age		
15–19 years	297	10.3
20–24 years	639	22.2
25–29 years	637	22.1
30-34 years	641	22.3
35 Years or Older	661	23.1
Race/Skin Color		
White	1004	35.5
Black	341	12.0
Mixed	1484	52.5
Living with a Partner		
Yes	2235	77.8
No	640	22.2
Per Capita Household Income		
≤ 1 MW**	2137	74.4
>1 MW**	737	25.6
Type of service		
Public	1974	70.3
Private	833	29.7
Health Professional (Most of the ANC Visits)		
Doctor	828	28.8
Non-Doctor	2043	71.2
Area of residence		
Urban	2425	84.3
Rural	450	15.7
Residing in a State Capital		
Yes	601	20.9
No	2273	79.1
Region of Residence		
North	340	11.8
Northeast	850	29.6
Southeast	1062	36.9
South	358	12.5
Center-West	265	9.2
Brazil	2875	100.0

Table 3 shows a positive and significant association between ANC adequacy and the educational level of the women: the higher the education, the better the adequacy of ANC. Around 30% of women with lower educational attainment have inadequate ANC. Regarding maternal age, better adequacy was observed among women aged 30 years or older. White women received higher-quality ANC compared to non-white women. ANC was more adequate among women living with a partner and those with a per capita household income greater than one minimum wage. ANC was also more adequate when provided in the private sector and when the healthcare provider was a physician. All sociodemographic and care-related factors showed a statistically significant association with ANC adequacy (p < 1%).  

**Table 3:** Proportional distribution (%) of pregnant women according to sociodemographic and antenatal care characteristics by antenatal care level of adequacy (Brazil, PNS-2019) χ2: Pearson's chi-square test; p*: P-value = descriptive significance level of the chi-square test for homogeneity of distributions; MW**: Minimum Wage in Brazil.

Variable	Antenatal Care Adequacy																		
	Inadequate				Level 1					Level 2					Level 3				
	n	%	χ2	p*		n	%	χ2	p*		n	%	χ2	p*		n	%	χ2	p*	
Level of Education			151.3	0.000				149.8	0.000				166.2	0.000				128.9	0.000	
Up to Elementary School	203	36.3				356	63.7				190	34.0				148	26.5			
Up to Junior High School	178	30.2				411	69.8				247	42.0				202	34.4			
Up to Senior High School	234	19.6				964	80.4				693	57.8				569	47.5			
Complete College Education	42	7.9				487	92.1				360	68.1				299	56.5			
Maternal Age			81.0	0.000				79.9	0.000				95.9	0.000				85.5	0.000	
15–19 years	108	36.2				190	63.8				91	30.5				77	26.0			
20–24 years	195	30.5				444	69.5				288	45.1				223	34.9			
25–29 Years	137	21.5				500	78.5				340	53.4				262	41.2			
30–34 years	106	16.5				536	83.5				374	58.3				307	47.8			
35 Years or Older	112	16.9				549	83.1				398	60.2				349	52.9			
Race/skin color			22.0	0.033				22.2	0.000				27.6	0.000				26.9	0.000	
White	194	19.3				810	80.7				570	56.7				479	47.7			
Black	108	31.6				233	68.4				138	40.5				110	32.3			
Mixed	343	23.1				1141	76.9				763	51.4				611	41.2			
Living with a partner			62.0	0.000				61.1	0.000				21.2	0.000				32.0	0.000	
Yes	437	19.6				1798	80.4				1210	54.1				1010	45.2			
No	219	34.3				420	65.7				280	43.8				209	32.6			
Per capita household income			92.6	0.000				92.0	0.000				85.8	0.000				75.7	0.000	
≤ 1 MW**	582	27.2				1555	72.8				1000	46.8				805	37.7			
>1 MW**	74	10.1				663	89.9				491	66.5				413	56.1			
Type of service			107.0	0.000				108.5	0.000				67.8	0.000				105.0	0.000	
Public	510	25.8				1464	74.2				952	48.2				735	37.2			
Private	83	10.0				750	90.0				537	64.4				482	57.9			
Health professional (most of ANC visits)			100.4	0.000				99.2	0.000				98.3	0.000				131.2	0.000	
Doctor	366	17.9				1680	82.1				309	37.3				214	25.8			
Non-Doctor	291	35.1				538	64.9				1181	57.7				1005	49.1			
Brazil	657	22.8				2218	77.2				1490	51.8				1219	42.4			

Also in Table 3, the distribution of each sociodemographic variable category is presented according to the levels of ANC adequacy (Inadequate, Level 1, Level 2, and Level 3). For all categories of sociodemographic variables, a decreasing trend is observed as the criteria for adequacy become stricter-that is, higher proportions are seen at Level 1 adequacy, while the lowest proportions are at Level 3. The same pattern was observed for ANC-related variables.  

Regarding the analysis of geographic characteristics (Table 4), women living in urban areas received more adequate ANC than those in rural areas (p < 1%) but living in a state capital was not significantly associated with the outcome. Among the macro-regions, the highest proportions of ANC adequacy were observed among women residing in the Southeast and South regions, while the lowest were among those living in the North, where 32.9% have inadequate ANC. These regional differences were statistically significant (p < 1%).  

**Table 4:** Proportional distribution (%) of pregnant women according to geographic characteristics by antenatal care level of adequacy (Brazil, PNS-2019) χ2: Pearson's chi-square test; p*: P-value = descriptive significance level of the chi-square test for homogeneity of distributions.

Variable	Antenatal Care Adequacy															
	Inadequate				Level 1				Level 2				Level 3			
	n	%	χ2	p*	n	%	χ2	p*	n	%	χ2	p*	n	%	χ2	p*
Area of Residence			25.3	0.000			25.3	0.000			61.4	0.000			48.1	0.000
Urban	513	21.2			1912	78.8			1333	55.0			1095	45.1		
Rural	144	31.9			306	68.1			157	34.9			124	27.5		
Residing in a state capital			0.8	0.445			0.8	0.362			0.1	0.007			0.1	0.802
Yes	129	21.5			472	78.5			316	52.5			252	41.9		
No	528	23.2			1746	76.8			1175	51.7			966	42.5		
Region of Residence			41.7	0.000			41.2	0.000			73.5	0.000			106.4	0.000
North	112	32.9			228	67.1			142	41.8			99	29.2		
Northeast	220	25.9			630	74.1			364	42.8			273	32.1		
Southeast	187	17.6			875	82.4			634	59.7			543	51.1		
South	76	21.2			282	78.8			207	57.9			182	50.9		
Center-West	62	23.4			203	76.6			143	54.1			122	45.9		
Brazil	657	22.8			2218	77.2			1490	51.8			1219	42.4		

A similar pattern was observed for geographic characteristics across adequacy levels, with higher proportions at Level 1 and lower at Level 3 (Table 4). It is worth noting the particularly low proportions (around 30%) of women in the North and Northeast regions who received the highest level of ANC adequacy, while in the Southeast and South, these proportions were approximately 50%. In Brazil, 77.2% of pregnant women receive quality ANC at Level 1, 51.8% at Level 2, and 42.4% at Level 3.  

**Figure 1:**
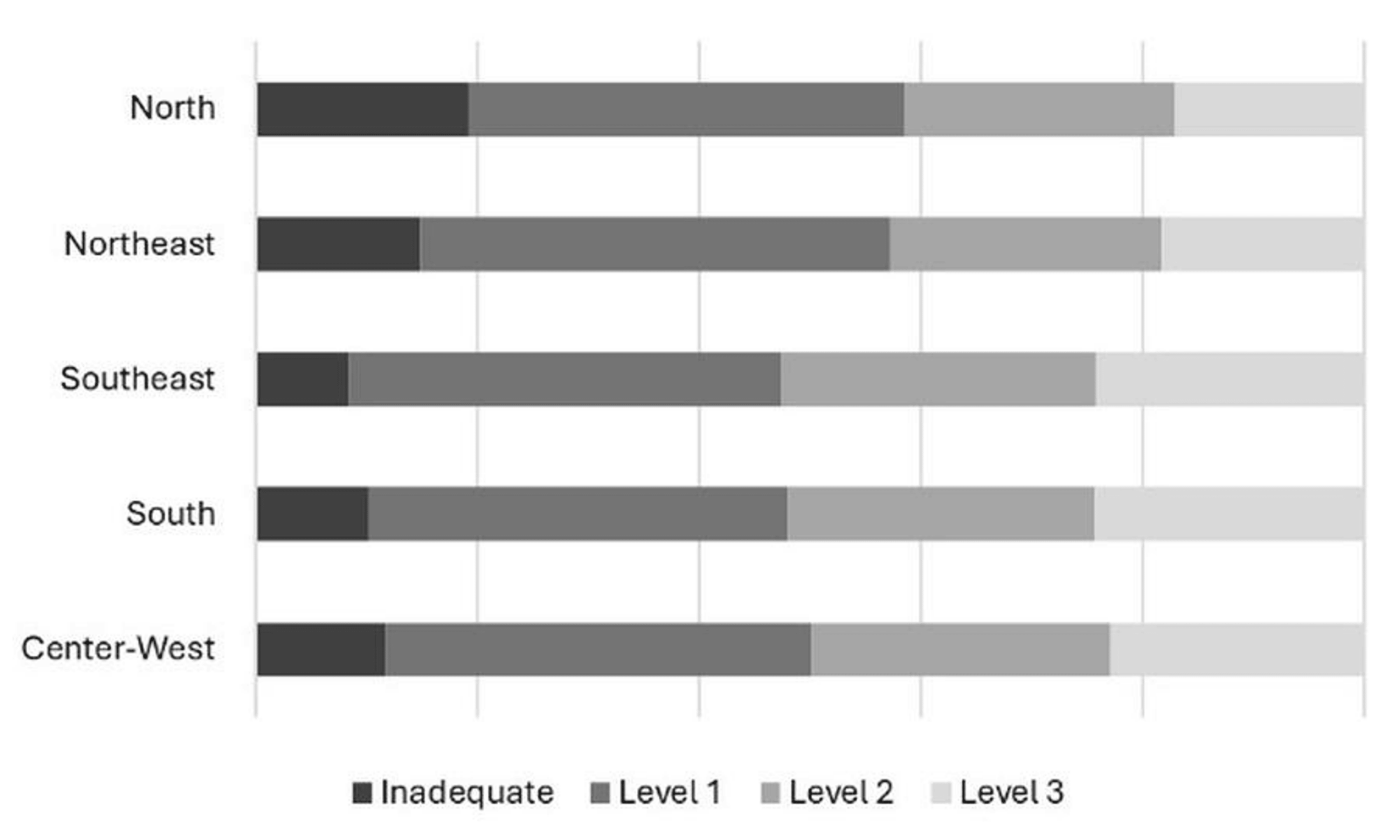
Regional differences in ANC adequacy (Brazil, PNS-2019)

In Figure 1, we presented a bar graph to visually depict regional disparities in ANC adequacy, where the dark gray bar represents the proportion of inadequacy and the lighter one the proportion of the highest level of adequacy. 

Table 5 presents the results of the multivariate logistic regression model, with the outcome being the highest level of ANC adequacy (Level 3). The sample size was 1219. Women residing in the North and Northeast regions had lower odds of receiving adequate ANC compared to those in the South (OR = 0.60 and OR = 0.65, 95% CI 0.39 - 0.93 and 0.44 - 0.95, respectively; p < 5%). Women living in urban areas compared to rural areas (OR = 1.64; 95% CI 1.20 - 2.26, p < 1%), as well as those with a partner or spouse (OR = 1.41; 95% CI 1.04 - 1.91, p < 5%), had significantly higher odds of receiving adequate ANC. Regarding education, women with elementary education had the lowest ANC adequacy (OR = 0.56; 95% CI 0.35 - 0.88, p < 5%). In terms of age, younger women were less likely to receive adequate ANC. As for ANC indicators, being attended by a physician was significantly associated with better adequacy (OR = 1.72; 95% CI 1.29 - 2.29, p < 1%). The remaining variables (living in a state capital, race/skin color, and location of consultations) were not statistically significant.  

**Table 5:** Results of the multivariate logistic regression model: association of sociodemographic, antenatal care and geographic characteristics with adequacy of/antenatal care at level 3 (Brazil, PNS-2019) *OR: Odds ratio; 95% CI: 95% confidence interval; p1-value: Descriptive significance level of the association of each factor with adequacy of antenatal care (level 3); NS: not significant.

Variable	OR*	95% CI	p1
Level of Education			
Up to Elementary School	0.56	0.35 – 0.88	0.011
Up to Junior High School	0.82	0.52 – 1.30	NS
Up to Senior High School	1.05	0.74 – 1.49	NS
Complete College Education	1.00	-	-
Maternal Age			
15–19 years	0.51	0.30 – 0.88	0.016
20–24 years	0.57	0.40 – 0.82	0.003
25–29 years	0.63	0.44 – 0.90	0.010
30-34 years	0.76	0.53 – 1.09	NS
35 years or older	1.00	-	-
Race/Skin color			
Black	0.74	0.47 – 1.15	0.178
Mixed	1.19	0.90 – 1.57	0.213
White	1.00	-	-
Living with a partner			
Yes	1.41	1.04 – 1.91	0.027
No	1.00	-	-
Type of service			
Private	1.31	0.94 – 1.84	NS
Public	1.00	-	-
Health professional (most of ANC visits)			
Doctor	1.72	1.29 – 2.29	0.000
Non-Doctor	1.00	-	-
Residing in a state capital			
No	0.78	0.59 – 1.03	NS
Yes	1.00	-	-
Area of Residence			
Urban	1.64	1.20 – 2.26	0.002
Rural	1.00	-	-
Region of Residence			
North	0.60	0.39 – 0.93	0.022
Northeast	0.65	0.44 – 0.95	0.027
Southeast	0.95	0.65 – 1.40	NS
Center-West	0.93	0.61 – 1.42	NS
South Region	1.00	-	-

## Discussion

Although the proportions of pregnant women who had six or more ANC visits and began ANC in the first trimester have reached high levels, 98.1% and 88.8%, respectively [5], the results of the present study show still low rates of adequate care, especially among women with precarious socioeconomic conditions. These findings are consistent with those of other studies, indicating that the quality of ANC has not advanced at the same pace as its coverage and is often linked to social inequities [25]. As previously noted, improving the quality of care is a slower process than expanding coverage [26]. Thus, even after more than 20 years of initiatives and numerous advances, the quality of ANC in Brazil remains a challenge to be overcome [2].  

The findings of this study showed that ANC tends to be less adequate in the North and Northeast regions of the country. In these regions, especially the North, which includes the Amazon region, the use of health services is lower due to difficulties in access [27]. In addition to stark geographic inequalities, factors such as low education, young maternal age, and absence of a partner were also associated with inadequate ANC. This situation is concerning, as the quality of ANC is directly associated with better perinatal outcomes [12].  

Low educational attainment is widely recognized as one of the main determinants of inadequate ANC. Firstly, it is linked to a lack of knowledge about the importance of ANC in preventing and/or detecting maternal and fetal conditions at an early stage, which enables healthy fetal development and reduces risks for the mother. Additionally, low maternal education is associated with other adverse factors, such as low income, difficulties in accessing health services, distance to facilities, and transportation costs [28]. 

As found in this study, lower adequacy of ANC among women aged 18 to 24 has been previously reported. Young women and/or adolescents, especially those in socially vulnerable situations, tend to start ANC late and attend fewer visits, which limits access to essential tests and makes adherence to care more difficult. The late initiation of care in this group limits the prevention of pregnancy complications such as chronic diseases, urinary tract infections, congenital syphilis, and vertical transmission of HIV infection [29].  

Regarding the lack of a partner, qualitative research has shown that partner abandonment after the woman becomes pregnant and lack of partner support are barriers to the use of prenatal care services. Conversely, those who receive psychological support from their partners are encouraged to attend ANC services and are more likely to adhere to subsequent follow-up services [30].

One of the strengths of our study is that it is based on a probabilistic sample, which allows for the estimation of natural expansion factors and, consequently, the sample is representative of the Brazilian population of pregnant women (from July 2017 to July 2019). Another strength is that the 2019 National Health Survey (PNS-2019) enabled us to investigate the progress made since 2013 and will allow us to examine the advances in ANC quality expected to be achieved by 2025-2026, when the third edition of the PNS will be conducted.

Other studies on the quality of ANC have been conducted previously, such as the “Birth in Brazil” study. In addition to prenatal care, this study includes results from clinical and obstetric examinations performed during consultations, laboratory tests, and diagnoses of diseases or complications during pregnancy, offering a greater level of detail than the PNS. However, the “Birth in Brazil” study is based on a cluster sampling design composed of hospitals with 500 or more live births in 2007. Since women who gave birth in hospitals with fewer than 500 live births and those who did not have a hospital delivery were not included in the survey, the sample is only representative of a subset of pregnant women [31]. Furthermore, the results of the second survey “Birth in Brazil II” are still not available.

Many other countries have also documented inequalities in ANC. The 2022-23 Demographic and Health Survey in Mozambique reported results similar to those found in Brazil. Use of healthcare during pregnancy showed significant disparities, favoring wealthier women over poorer ones and urban residents over those in rural areas. Besides distance to the nearest healthcare unit, and the province of residence, exposure to the media (TV/radio/newspapers) was another determining factor, encouraging health-seeking behaviors and filling knowledge gaps. In addition to traditional media, the authors propose disseminating related information through the internet, social media, or other easily accessible channels [32].  

A survey conducted in Tanzania showed that key factors for adequate ANC included giving birth in healthcare facilities and scheduling appointments via the internet or mobile phone, highlighting the need to promote internet access to facilitate ANC appointment booking [33]. A study in Australia identified the main barriers to receiving timely and appropriate ANC. For many interviewed women, social vulnerability, lack of information, and problems in healthcare delivery, such as inflexible hours and difficulty accessing services, were major challenges [34]. In Brazil, the use of technologies such as cell phones and the Internet to schedule ANC appointments is still limited, although it could be a valuable tool to improve communication between pregnant women and health teams.  

In Brazil, even among women with higher education levels and/or those receiving ANC in private healthcare services, the proportion achieving high-level adequacy did not reach 60%. In other words, the problem of accessing quality ANC is not solely due to socioeconomic conditions, but also the quality of care provided [35]. A study conducted in two municipalities in the Northeast region showed that, in addition to socioeconomic hardship and access difficulties among women living in rural communities, the main issues were the absence of medical equipment, delays or failures in conducting exams and measurements on mothers and babies [36].  

According to SUS principles, access to quality healthcare during pregnancy and childbirth is a fundamental right for women. However, in the case of reproductive health, there is a gap between the legal rights of women and what the healthcare system actually provides [35,36]. The findings here depicted emphasize the importance of monitoring the quality of prenatal care, especially to identify and meet the needs of the most vulnerable women during pregnancy, providing support for public ANC policies. 

Limitations  

The National Health Survey (PNS), by employing trained interviewers capable of using appropriate approaches and communication with participants, offers the advantage of collecting detailed information about procedures performed during ANC. Nevertheless, the use of self-reported data obtained through home interviews is a limitation, as it may be subject to recall bias, overreporting of procedures to match the expected response, or underreporting due to difficulties in understanding the questions or discomfort in responding to certain topics. In addition, since it was not possible to consider the gestational age at birth, premature babies may not have met the criteria for ANC adequacy due to the short gestation period. Another limitation is the inability to analyze certain aspects of health care delivery, such as the qualifications of health teams, the hours of service, and the barriers encountered in accessing ANC services.  

Non-measurable factors from PNS data, such as healthcare facility infrastructure, availability of resources, and professional training, can significantly affect assessments of prenatal care quality and coverage. These factors, which were not addressed in the study, can directly influence the quality of care provided. Women treated in environments with fewer resources or poorly trained teams may receive inadequate care and become discouraged from attending subsequent appointments. The lack of medical equipment and supplies in primary care units in Brazil certainly restricts the scope of actions, limiting the possibilities of providing quality prenatal care [37]. 

## Conclusions

This study highlights that adequate ANC remains a persistent challenge in Brazil, despite over two decades of public policies aimed at improving healthcare services. While the number of consultations and early initiation of ANC have improved significantly and equitably, the quality of care continues to reflect the country’s entrenched social and regional disparities. Understanding these inequalities is essential for guiding more effective and equitable public health strategies, particularly in identifying and addressing the needs of the most vulnerable groups. 

Ensuring access to quality ANC requires integrated actions such as strengthening links with referral maternity hospitals, promoting health education, and expanding access to information, especially regarding pregnant women's rights. Health managers and ANC teams must be prepared to mitigate the ANC quality disparities shown in the present study, focusing on the most vulnerable groups, such as women with low educational attainment, adolescents, and residents of the North and Northeast regions living in precarious socioeconomic conditions. Key challenges include insufficient sustainable financing for quality ANC, limited access to information, and barriers to reaching health services, particularly for those most in need. To ensure inclusion, equity must be central to all planned interventions, guaranteeing dignified, timely, and high-quality care for every pregnant woman. As children are the foundation of Brazil’s future, universalizing child health from birth must be viewed as a fundamental priority for sustainable development. 
